# Study on Preparation and Performance of CO_2_ Foamed Concrete for Heat Insulation and Carbon Storage

**DOI:** 10.3390/ma16072725

**Published:** 2023-03-29

**Authors:** Xupeng Ta, Yuan Zhang, Zhijun Wan, Peng Shi, Jiale Zhou

**Affiliations:** 1 School of Mines, China University of Mining & Technology, Xuzhou 221116, China; taxupeng@cumt.edu.cn (X.T.);; 2 Department of New Energy Science & Engineering, China University of Mining & Technology, Xuzhou 221116, China; 3 Key Laboratory of Deep Coal Resource Mining, Ministry of Education of China, China University of Mining & Technology, Xuzhou 221116, China

**Keywords:** foam concrete, carbon sequestration, coal and electricity integration, thermal conductivity

## Abstract

Environmental problems caused by large amounts of CO_2_ generated by coal–electricity integration bases have raised concerns. To solve these problems, this study develops a CO_2_ foam concrete (CFC) material with both heat insulation and carbon fixation characteristics to realize CO_2_ in situ storage and utilization. In this study, a Portland-cement-based CO_2_ foam concrete (PC-CFC) with good thermal insulation performance and carbon fixation ability is prepared using carbonation pretreatment cement and a physical foaming method. The effects of CO_2_ on the compressive strength, thermal insulation, and carbon fixation properties of PC-CFC are studied. The internal relationship between the compressive strength, thermal insulation, and carbon fixation performance of PC-CFC is analyzed, and the feasibility of PC-CFC as a filling material to realize the in situ mineralization and storage of CO_2_ in the coal–electricity integration base is discussed. The experimental results show that the compressive strength of PC-CFC is significantly improved by CO_2_ curing. However, CO_2_ in the PC-CFC pores may weaken the strength of the pore structure, and the compressive strength decreases by 3.62% for each 1% increase in PC-CFC porosity. Using CO_2_ as a foaming gas and the physical foaming method to prepare CFC can achieve improved thermal insulation performance. The thermal conductivity of PC-CFC is 0.0512–0.0905 W/(m·K). In addition, the compressive strength of PC-CFC increases by 19.08% when the thermal conductivity of PC-CFC increases by 1%. On the premise of meeting the thermal insulation requirements, PC-CFC can achieve improved compressive strength. The carbon sequestration rate of the PC-CFC skeleton is 6.1–8.57%, and the carbon storage capacity of PC-CFC pores is 1.36–2.60 kg/ton, which has obvious carbon sequestration potential; however, the preparation process and parameters of PC-CFC still require further improvement. The research results show that PC-CFC has great potential for engineering applications and is of great significance for realizing carbon reduction at the coal–electricity integration base.

## 1. Introduction

Coal and electricity integration plays an important role in ensuring national energy security; however, there is also an urgent need for carbon reduction. The main carbon emission source of the coal–electricity integration base is coal-fired power plants. Coal-fired power plants account for more than 30% of China’s total emissions and are the largest industrial fixed source of CO_2_ emissions in China [1]. At present, the CO_2_ treatment methods of the coal–electricity integration base are mainly geological storage, oil extraction, and gas production; however, problems such as high transportation costs and CO_2_ leakage from land pipelines, railways, and highway vehicular modes exist [2,3]. These problems urge the development of CO_2_ in situ storage and utilization technology for coal–electricity integration bases. Foam concrete is a bridge between CO_2_ in situ storage and mine filling needs. Foam concrete is the main material used in mine filling and has been widely applied in the filling and utilization of solid wastes, such as fly ash and steel slag [4,5,6,7,8,9,10]. CO_2_ foam concrete (CFC) is a porous material prepared with CO_2_ as the foaming gas and is a potential low-carbon energy-saving material. The expected carbon fixation value of CFC in China is estimated to reach 2.9 billion tons between 2017 and 2021 [11]. Therefore, the use of CO_2_ to prepare foam concrete for gob filling is expected to ease the difficulties of CO_2_ in situ storage and utilization.

The storage and utilization of CO_2_ by CFC are realized through the mineralization reaction of cement-based materials and pore carbon storage. On the one hand, the cementitious materials containing Ca^2+^ and Mg^2+^ in the CFC skeleton and their hydration products react with CO_2_ [11,12]. This mineralization reaction process can potentially be integrated into the production process of prefabricated CFC, and CO_2_ can be stored in cement-based materials in the form of inorganic carbonate. Furthermore, it can also effectively improve the mechanical properties and durability of CFC materials [13]. Currently, researchers promote the mineralization reaction of CO_2_ and cement-based materials by CO_2_ curing, mixing concrete, or adding CO_2_ into concrete in the form of dry ice, carbonate, and other additives [14]. The literature shows that under CO_2_ curing pressures of 0.2–44.82 MPa, curing times of 60–6000 min, and curing temperatures of 20–150 °C, the CO_2_ storage capacity of cement-based materials is in the range of 0.04–0.58 g/g [12,15,16]. On the other hand, the pores of CFC have certain carbon storage capacities, which may have been ignored by scholars. Each ton of CFC (porosity of 58.9%) can store 1.95 kg of additional carbon. Based on China’s cement production in 2020, the maximum carbon storage in CFC pores can reach 4.6 million tons [11]. It can be seen that compared with the traditional CO_2_ curing method, CFC has a noticeable carbon fixation potential.

In addition, compared with traditional foam concrete, CFC exhibits an improved thermal insulation performance. Previous studies have shown that foaming gas is one of the main factors affecting the thermal conductivity of foam concrete [17]. The thermal conductivity of CO_2_ gas is 0.015 W/(m·K), which is lower than that of air (0.023 W/(m·K)), oxygen (0.024 W/(m·K)), and nitrogen (0.024 W/(m·K)). Therefore, from the perspective of the thermal conductivity of foaming gas, it is expected that foam concrete materials with improved thermal insulation performance can be obtained by using CO_2_ as the foaming gas. Li et al. used CO_2_ as a foaming gas to prepare magnesium-phosphate-cement-based foam concrete, with a thermal conductivity ranging from 0.10 to 0.30 W/(m·K) [17]. In addition, Li also prepared sulfoaluminate-cement-based foam concrete with a thermal conductivity ranging from 0.052 to 0.13 W/(m·K) [18]. Ma et al. used CO_2_ as the foaming gas to prepare magnesium-phosphate-cement-based foam concrete with a thermal conductivity range of 0.066–0.341 W/(m·K) [19]. Currently, mining enterprises prevent the heat transfer of the surrounding rock to the roadway by spraying thermal insulation materials on the surface of the high-temperature roadway and injecting thermal insulation materials inside the surrounding rock of the roadway [20]. The thermal conductivity of the applied thermal insulation materials for mining is 0.15–0.40 W/(m·K) [21,22,23]. Comparing the thermal conductivities of the abovementioned thermal insulation materials, it can be seen that CFC materials have improved thermal insulation performance. It can be seen that CFC can be used for filling the gob to achieve CO_2_ storage and can also be used as a potential thermal insulation material.

The potential carbon fixation and thermal insulation characteristics of CFC have opened up for the sustainable development of the coal power industry; however, few scholars have conducted research in this area. This is owed to the high price of cementitious materials and chemical foaming agents used in the existing CFC preparation methods, which makes CO_2_ mineralized filling uneconomically feasible. In addition, there is a strong mineralization reaction between Portland cement commonly used for mine filling and CO_2_ foam, which leads to a poor foaming effect, making it difficult for scholars to successfully prepare Portland-cement-based CO_2_ foam concrete (PC-CFC). The abovementioned problems hinder further research on CO_2_ in situ storage and utilization technology and hinder the preliminary understanding of PC-CFC thermal insulation and carbon sequestration potential. In this study, PC-CFC was successfully developed through the carbonation pretreatment of cement and physical foaming. The effects of CO_2_ on the mechanical, thermal, and carbon fixation properties of PC-CFC and their mechanisms were studied. To the best of our knowledge, this is the first study to investigate the thermal insulation and carbon fixation performance of PC-CFC. Finally, the feasibility of using PC-CFC to replace traditional Portland cement foam concrete under certain working conditions is discussed to contribute to the low-carbon and efficient development of the coal power integration base.

## 2. Experiment Details

### 2.1. Materials

The strength grade of the Portland cement used (Jiuqi Building Materials Co., Ltd., Zhucheng, China) was 42.5 and its chemical composition is listed in Table 1. The initial and final coagulation times were 172 min and 234 min, respectively. The foaming gas used was CO_2_ (Special Gas Plant, Xuzhou, China) with a purity > 99.99%. Lauryldiethanolamine (C_16_H_35_NO_2_, AC-1202, produced by the Haian Petrochemical Plant, Haian, China) was the surfactant used, and the effective mass fraction was 99.5%. The foam stabilizer used was hydroxypropyl methylcellulose (C_12_H_20_O_10_, HPMC, produced by Qiyue Chemical Materials Co. Ltd., Jinan, China). Tap water was used for mixing when the temperature ranged from 14 to 20 °C.

### 2.2. Experimental Device and Sample Preparation

**Figure 1:**
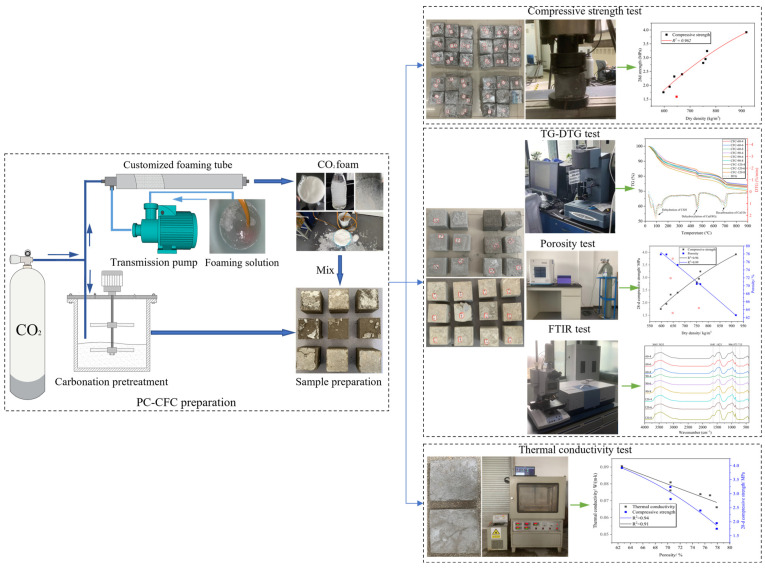
Experimental device and process.

The experimental device arrangement and experimental process are presented in Figure 1. CO_2_ significantly reduces the fluidity of Portland cement slurry under sealed conditions [24,25]. Therefore, this study conducted carbonation pretreatment of Portland cement slurry in an open environment. CO_2_ is slightly soluble in water, resulting in the short half-life of ordinary CO_2_ foam [26,27]. To ensure the stability of the CO_2_ foam, a foaming solution was prepared by mixing a surfactant, foam stabilizer, and water in a mass ratio of 1:2:500, respectively. In water, CO_2_ reacts with the mineral phase of Portland cement and its hydration products. However, a high water–cement ratio will hinder the mineralization reaction and a low water–cement ratio will affect the fluidity of the slurry. The water–cement ratio was determined to be 0.5, based on previous experiments. The mix compositions of PC-CFC are listed in Table 2. The specific experimental steps are as follows:

(1) Portland cement slurry: The water–cement ratio is known to affect the performance of CO_2_ foam concrete. Higher water–cement ratios are expected to lead to foam bursting and strength reduction, whereas lower water–cement ratios are expected to lead to a poor fluidity of cement slurries; moreover, the mixing of CO_2_ is more likely to reduce fluidity [24,25]. In this study, aiming to ensure the fluidity of cement slurry, the water–cement ratio of PC-CFC was determined to be 0.5 through multiple tests [13]. To obtain Portland cement paste, pre-weighed cement was poured into a mixing bucket; this was followed by the addition of a certain amount of water, followed by mixing for 180 s at a speed of 200 r/min and for 120 s at a speed of 110 r/min.
(2) Carbonation pretreatment: Portland cement subjected to carbonation pretreatment was obtained by mixing Portland cement slurry and CO_2_ in a mixer. The CO_2_ cylinder was connected to the mixing drum via a pressure pipe of diameter 5 mm, and the CO_2_ output pressure was set to approximately 0.10 MPa. In early CO_2_ curing research of cement-based materials, the CO_2_ pressure selected was typically between 0.10 and 0.50 MPa, which is beneficial in improving the mineralization reaction rate [28]. The agitator mixed the Portland cement slurry at 110 revolutions (rev)/min to obtain the Portland cement slurry after carbonation pretreatment.
(3) Physical foaming: CO_2_ foam is produced by fully mixing the foaming liquid and CO_2_ in a foaming device under a certain pressure. The foaming liquid was continuously sucked into a customized foaming machine using a suction pump. The customized foaming machine was connected to a CO_2_ cylinder via a pressure pipe with a diameter of 5 mm, and the CO_2_ output pressure was set in the range of 0.20–0.30 MPa. It is worth noting that the CO_2_ output pressure must be matched with the liquid absorption speed of the foaming device to obtain the CO_2_ foam in a stacked state.
(4) PC-CFC sample preparation: Sample preparation was conducted simultaneously with the preparation of the CO_2_ foam. The carbonated pretreated cement paste was weighed (500 g) and mixed with CO_2_ foam. The mixture was then injected into the mold until it was full. After 48 h, the samples were removed from the molds and cured with relative humidity (RH) > 95% at 20 ± 2 °C for 28 days (d). It is worth noting that during foam concrete preparation, 24 h demolding is often adopted [10]. However, owing to the damage caused to the CO_2_ foam and the promotion of hydration reactions by PC-CFC, the demolding of samples with high water contents within 24 h is very likely to cause sample damage; hence, demolding after 48 h was selected here.

### 2.3. Test Methods

#### 2.3.1. Compressive Strength and Dry Density

The PC-CFC samples with 28 d curing were tested for compressive strength at a loading rate of 0.5 MPa/min (Table 3). The average data from three independent tests were used to obtain the final compressive strength.

The PC-CFC sample with 28 d curing was dried to a constant weight at 105 °C (Table 3). The sample size was measured using a Vernier caliper, and the average of the three measurements was used to minimize errors. The average data from the three independent tests were used to obtain the dry density.

#### 2.3.2. Porosity

Porosity was measured using an automatic true density and porosity analyzer (BSD-TD-K). The adsorbed gas was helium, and the gas output pressure was approximately 0.4 MPa. The test sample was acquired from the PC-CFC sample after testing the dry density. To meet the requirements of the sample bin, the test sample was processed into a cylinder with a radius of approximately 5 mm and a height of approximately 30 mm for testing (Table 3).

#### 2.3.3. Thermal Conductivity

The thermal conductivity was measured using a plate heat flow meter (DRPL-400). The fabricated specimen was a 300 mm × 300 mm × 30 mm flat test piece to be placed in a drying oven (60 ± 5 °C) after curing for 28 d to dry to a constant mass for testing (Table 3).

#### 2.3.4. Fourier Infrared Spectrum Analysis (FTIR)

FTIR was performed using an infrared spectrometer (VERTEX-80V). The wavenumbers ranged from 4000 to 400 cm^−1^. The test sample was acquired from the PC-CFC sample after testing the dry density. The PC-CFC sample was ground using an agate mortar, and the ground sample was passed through a square-hole sieve (size: 45 μm) to obtain the powder sample (Table 3).

#### 2.3.5. Thermogravimetric Analysis (TG)

The TG spectra were measured using a thermal analyzer (TA-Q500). The temperature was increased from 30 °C to 900 °C at a heating rate of 15 °C/min under a nitrogen atmosphere. The acquisition method for the test sample was the same as that for the FTIR test sample (Table 3). CO_2_ uptake was calculated using the following equation [6,29]:

(1) $${\mathrm{CO}}_{2}\;\mathrm{uptake}\left[\mathrm{wt}.\;\%\right]=\frac{\Delta W_{540\text{–}800\;℃}}{\Delta W_{105\;℃}}$$

## 3. Results and Discussion

### 3.1. Compressive Strength

**Figure 2:**
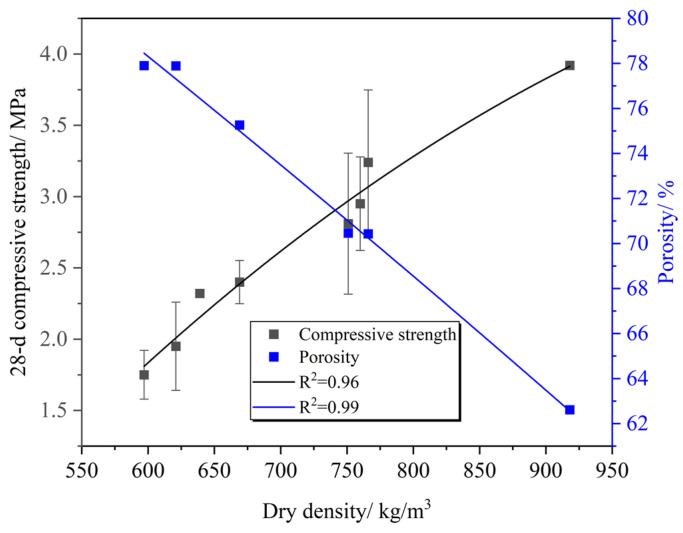
Relationship between the 28 d compressive strength and porosity and dry density.

The test results of PC-CFC are listed in Table 4. Figure 2 shows the relationship between the PC-CFC 28 d compressive strength and porosity. It can be observed that the 28 d compressive strength of PC-CFC gradually decreased with an increase in porosity. The porosity increased from 62.61% to 77.9%, whereas the 28 d compressive strength decreased by 3.62% when the porosity increased by 1%, and the strength of Portland cement foam concrete decreased by approximately 2.6–2.8% when the porosity increased by 1% [30]. The PC-CFC strength attenuation amplitude was higher than that of the Portland cement foam concrete. Two possible reasons for this phenomenon are as follows: first, with the increase in porosity, the pore wall becomes thinner, and the PC-CFC skeleton gradually becomes sparse, resulting in strength attenuation; second, there is a weakening effect of CO_2_ in the pores. A curing environment with high relative humidity and high CO_2_ concentration in the region may be formed in the pores. Research shows that this environment leads to the production of a large amount of aragonite, and the strength of aragonite is lower than that of calcite, resulting in a reduction in pore strength [31]. SEM testing indicates that aragonite is indeed produced around the pores of PC-CFC [13]. Under the combined action of these two factors, the strength attenuation amplitude of PC-CFC is greater than that of Portland cement foam concrete.

**Figure 3:**
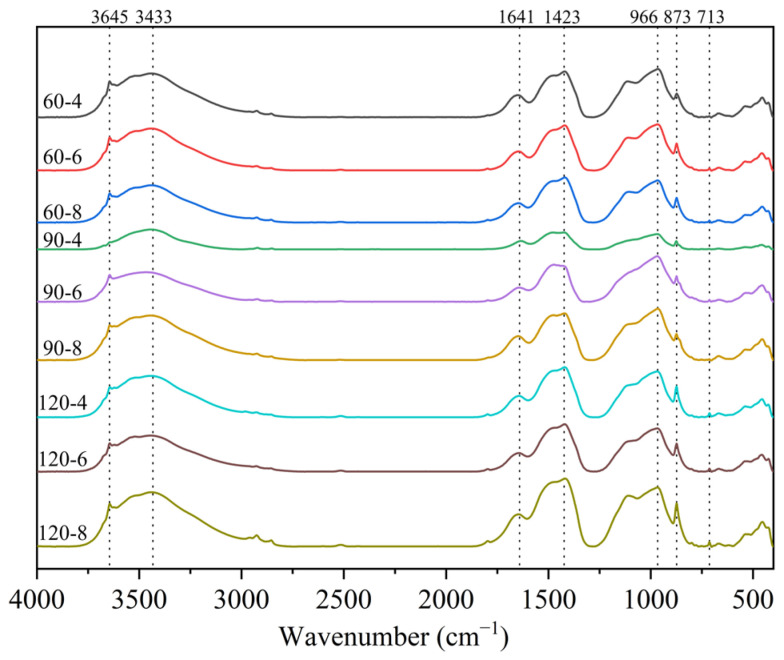
FTIR spectra of PC-CFC.

Another important characteristic observed in Figure 2 is the relationship between the PC-CFC dry density, 28 d compressive strength, and porosity. It can be seen that with an increase in dry density, the compressive strength gradually increased and the porosity gradually decreased. There was a negative correlation between compressive strength and porosity. The PC-CFC exhibited improved compressive strength. Taking a sample with a dry density of approximately 639–669 kg/m^3^ as an example, the 28 d compressive strength of PC-CFC was in the range of 1.59–2.40 MPa. In previous studies, the 28 d compressive strength of Portland cement foam concrete with a dry density of approximately 650 kg/m^3^ has been reported to be 0.5–1.2 MPa [19,32]. The 28 d compressive strength of PC-CFC prepared in this study was twice that of Portland cement foam concrete. Fourier infrared spectrum analysis was performed to clarify the cause of this phenomenon. Figure 3 shows the FTIR spectrum of the CFC (4000–400 cm^−1^). As shown in Figure 3, the absorption peaks near the wavenumbers of 713 cm^−1^, 875 cm^−1^, 1419 cm^−1^, and 1474 cm^−1^ correspond to calcium carbonate formed by the carbonation reaction of calcium silicate minerals with CO_2_ [33,34]. In addition, the absorption peaks near 3440 cm^−1^ correspond to the OH^−^ vibration peak in the adsorbed water [35,36]. There is a clear calcium carbonate absorption peak at 713 cm^−1^ in the PC-CFC as shown in the figure. According to the literature on Portland cement after curing [37], it can be seen that this is the calcium carbonate absorption peak formed by carbonation pretreatment. The above phenomenon shows that the curing effect of carbonation pretreatment on PC-CFC results in a higher 28 d compressive strength.

**Figure 4:**
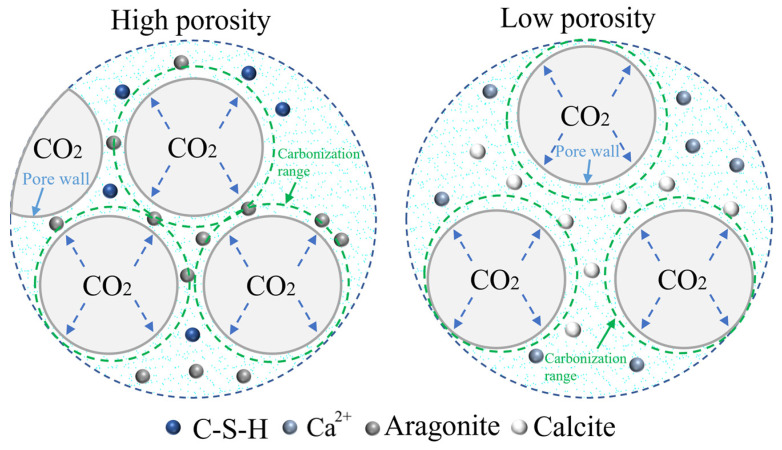
Effect of CO_2_ on the hole wall structure under different porosities.

In order to further understand the effect of CO_2_ on the pore wall structure, Figure 4 shows the effect of CO_2_ on the pore wall structure under various porosities. Generally, in a relatively low humidity environment, CO_2_ primarily reacts with Ca(OH)_2_ to form calcite [38,39]; however, in a relatively high humidity environment, CO_2_ primarily reacts with C-S-H to form aragonite [31]. In the case of high porosity, the distance between pores is compact, the pore wall structure is affected by CO_2_ in several pores, and the pore liquid film provides an environment with high relative humidity. In the pore area, aragonite is primarily formed through a carbonation reaction between C-S-H and CO_2_. At this time, CO_2_ curing plays a role in weakening the pore wall structure. In the case of low porosity, there is a certain distance between the pores. A small amount of CO_2_ inside the pores can only form a curing effect on the calcium silicate mineral phase and hydration products around the pore wall. The carbonation reaction between Ca(OH)_2_ and CO_2_ in the pore area primarily forms calcite. At this time, CO_2_ curing plays a role in strengthening the pore wall structure.

In typical underground mining operations, to ensure the safety of underground operations, the compressive strength of the filling body must reach 0.7–2.0 MPa after 28 d to provide good roof support [5,40,41]. The strength of the PC-CFC meets the backfill requirements of the gob; however, its strength is still low, which makes it difficult to meet the requirements of underground structural engineering. Therefore, the preparation process for PC-CFC still requires further improvement to meet the requirements for higher use.

### 3.2. Thermal Conductivity

**Figure 5:**
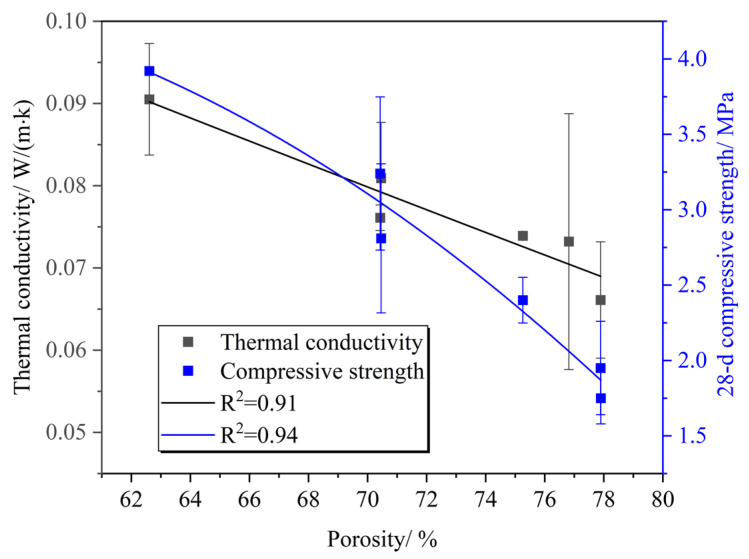
Relationship between the thermal conductivity and 28 d compressive strength and porosity.

The relationship between the thermal conductivity and porosity of PC-CFC is shown in Figure 5. It can be observed that the thermal conductivity of PC-CFC decreased gradually with an increase in porosity. Previous studies have shown that the thermal conductivity of Portland cement foam concrete decreases with increasing porosity [30]. The influence of porosity on the thermal conductivity of PC-CFC and Portland cement foam concrete was consistent. The porosity increased from 62.61% to 77.9% and the thermal conductivity of PC-CFC decreased by 43%. The thermal conductivity of PC-CFC decreased by 2.81% for each 1% increase in porosity. In foam concrete with oxygen foaming and CO_2_ curing, the thermal conductivity decreased by 1.06% [30] for every 1% increase in porosity, which is smaller than that of PC-CFC. The reason for this phenomenon is that the inside of the PC-CFC pore is CO_2_, and the thermal conductivity of carbon dioxide (0.0143 W/(m·K)) is less than that of oxygen (0.0240 W/(m·K)); thus, the thermal conductivity of PC-CFC decreases significantly. This shows that PC-CFC prepared with CO_2_ as the foaming gas has an enhanced thermal insulation performance.

Figure 5 also shows the relationship between the compressive strength and thermal conductivity of the PC-CFC. With an increase in the 28 d compressive strength, the thermal conductivity increased gradually. In the application of thermal insulation materials, it is often anticipated that the materials will have improved thermal insulation performance and higher compressive strength. The thermal conductivity and compressive strength of the thermal insulation materials used in mines and previously prepared by our laboratory increased by 1% and 2.76% [23], whereas the thermal conductivity and compressive strength of PC-CFC increased by 1% and 19.08%, respectively. Therefore, PC-CFC can achieve improved compressive strength on the premise of meeting thermal insulation requirements. The reason for this trend is that CO_2_ can strengthen PC-CFC through carbonization curing and also reduce the pore thermal conductivity to strengthen the thermal insulation performance of PC-CFC. However, the strength of the materials required by mines is higher than that of C10 concrete, which means that PC-CFC can only be used for mine thermal insulation systems. If PC-CFC is to be used in mine structural engineering, the coordination between its strength, thermal insulation, and porosity requires further optimization.

**Figure 6:**
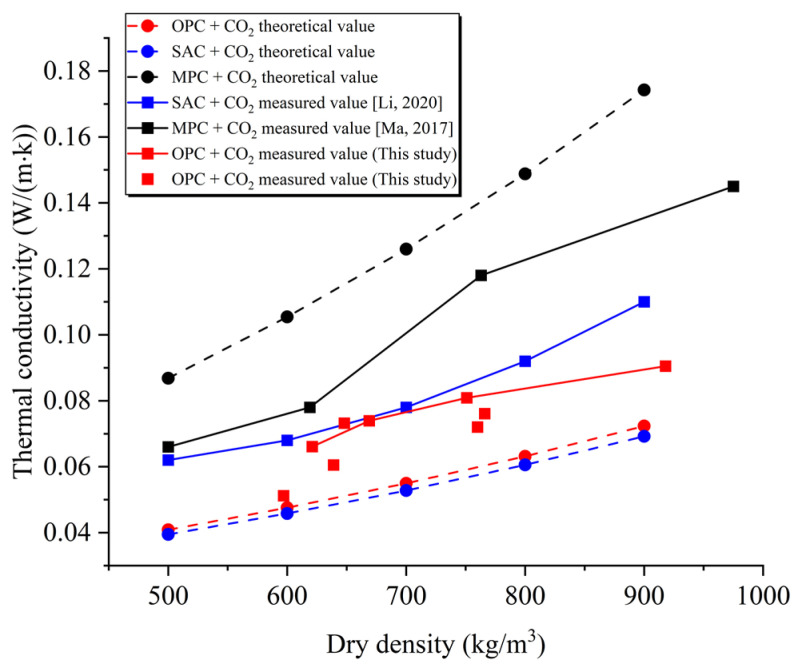
Distribution law of the thermal conductivity of CFC [17,19].

To further understand the thermal insulation performance of PC-CFC, we compared the difference between the theoretical and measured thermal conductivities of magnesium phosphate cement (MPC) foam concrete, sulfoaluminate cement (SAC) foam concrete, and PC-CFC. The calculation method for the thermal conductivity of foam concrete is as follows [17]:

(2) $$\lambda =\frac{\left(\lambda_{2}-\lambda_{1}\right)\rho_{1}}{\left(5\rho_{2}-4\rho_{1}\right)z}+\lambda_{1}$$

where λ is the thermal conductivity of foam concrete; $\lambda_{1}$ and $\lambda_{2}$ are the thermal conductivities of the foaming gas and cement slurry, respectively; $\rho_{1}\;$and $\rho_{2}$ are the dry densities of foam concrete and cement, respectively; z is the ratio of closed pores of foam concrete to all pores. When all pores were closed, z was equal to 1. The dry density of the Portland cement used was 2100 kg/m^3^, and the thermal conductivity was approximately 0.4544 W/(m∙K). Other related parameters were obtained from previous studies [17]. Assuming that all pores are closed and the pore structure of all foam concretes prepared with cement is the same, the theoretical value of the thermal conductivity of CFC prepared with different types of cement is shown in Figure 6.

Figure 6 shows the distribution law of the theoretical and measured values for various cement-based CFCs. With an increase in density, the theoretical value of the thermal conductivity of all foam concretes gradually increased. The theoretical values of the thermal conductivities of PC-CFC and SAC foam concrete were similar, and both were smaller than the theoretical values of the thermal conductivity of MPC foam concrete. The reason for this phenomenon is that the thermal conductivity of Portland cement slurry is 0.4544 W/(m∙K), which is approximately the same as that of the SAC slurry (0.4307 W/(m·K)) and approximately 1/3 that of the MPC slurry (1.236 W/(m·K)).

In the experimental study, the measured value of PC-CFC thermal conductivity (0.0512–0.0905 W/(m·K)) was equivalent to that of SAC foam concrete’s thermal conductivity (0.062–0.110 W/(m·K)), and was significantly lower than the measured value of MPC foam concrete (0.063–0.144 W/(m·K)), as shown in Figure 6. The distribution trend of this measured result was approximately the same as that of the theoretical calculation. It should be noted that the measured value of PC-CFC was slightly less than the measured value of SAC foam concrete. The reason for this difference could be that the foaming methods used in the two experiments were different. Compared to the chemical foaming method used in the preparation of MPC foam concrete, the physical foaming method used in this study has the characteristics of uniform foam and controllable pore size. CFCs with uniform bubble distributions and small pore sizes tend to have low thermal conductivities [42]. In addition, it was noted that the measured value of PC-CFC was slightly larger than the theoretical value, and the reason for this difference is that CO_2_ mineralization or escape might exist in the pores, and the gas in the pores was replaced with air of higher thermal conductivity, resulting in a thermal conductivity that is higher than the theoretical value. The above research results show that the preparation of PC-CFC by the physical foaming method using Portland cement as the cementing material can achieve good thermal insulation performance and assist in controlling the cost.

### 3.3. CO_2_ Uptake

**Figure 7:**
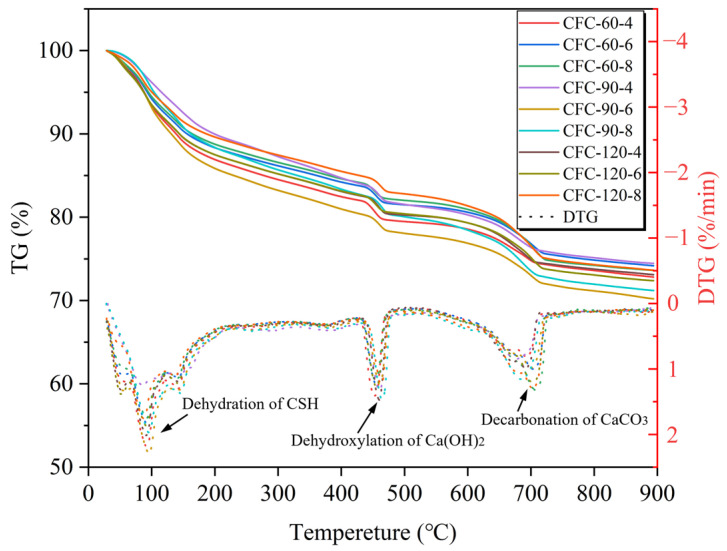
TG-DTG of PC-CFC.

**Figure 8:**
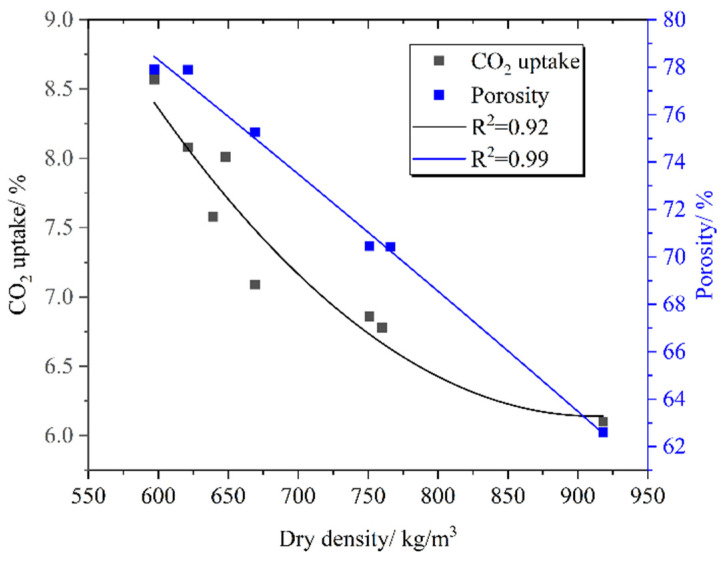
Relationship of CO_2_ uptake and porosity to dry density.

Figure 7 shows the thermogravimetric curve of PC-CFC. The main weight loss temperatures were located at 100 °C, 480 °C, and 700 °C. Previous studies have shown that C-S-H gels dehydrated at temperatures 100–400 °C, Ca(OH)_2_ dehydrogenated at temperatures 400–480 °C [43], and CaCO_3_ was decomposed at temperatures in the range of 540–800 °C. Figure 8 shows the relationship between the carbon fixation rate, porosity, and density of PC-CFC. It can be seen that with an increase in density, the carbon fixation rate and porosity of PC-CFC gradually decreased. Two reasons for this reduction are as follows: the reduction in carbonation pretreatment time in the preparation of PC-CFC will lead to the damage of foam and the escape of CO_2_, whereas the reduction in carbonation pretreatment time and foam amount will lead to the reduction in CO_2_ input, resulting in the reduction in carbon sequestration of PC-CFC.

The carbon storage capacity of PC-CFC pore can be calculated by pore volume, which can be estimated from porosity. It is accurate to use porosity as the foundation for computation because a small amount of CO_2_ might also be present in skeleton pores in PC-CFC. The porosity of PC-CFC is 62.61–77.9%, and its pore volume per ton is 0.68–1.30 m^3^. If all the pores contain CO_2_ gas at 1 atmospheric pressure, the carbon storage capacity of PC-CFC pore is 1.36–2.60 kg/ton (the density of CO_2_ is 1.997 kg/m^3^). This is equivalent to saving 0.75–1.44 m^3^ of natural gas or 1.71–3.27 kW·h of electricity. According to statistics, the total production of foam concrete in China is about 60 million m^3^ in 2020 [11], and the carbon storage capacity of foam concrete pores (porosity is 77.9%) can reach 93,300 tons.

**Figure 9:**
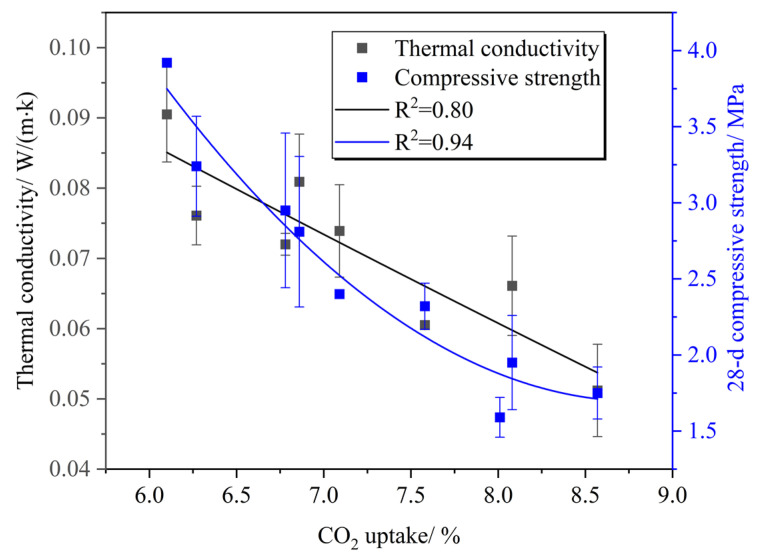
Relationship between the thermal conductivity and 28 d compressive strength and CO_2_ uptake.

Figure 9 shows the relationship between the carbon fixation rate, 28 d compressive strength, and thermal conductivity. It can be seen that with an increase in the carbon fixation rate, the thermal conductivity of PC-CFC decreased and the 28 d compressive strength decreased gradually. This means that PC-CFC can achieve good thermal insulation and carbon fixation performance on the premise of meeting the strength requirement of the mine filling body (0.7–2 MPa). This feature enables PC-CFC to permanently seal CO_2_ in the gob and also to block the heat returned from the gob. This indicates that PC-CFC is expected to become a new functional material with good thermal insulation properties and carbon fixation capacity.

**Figure 10:**
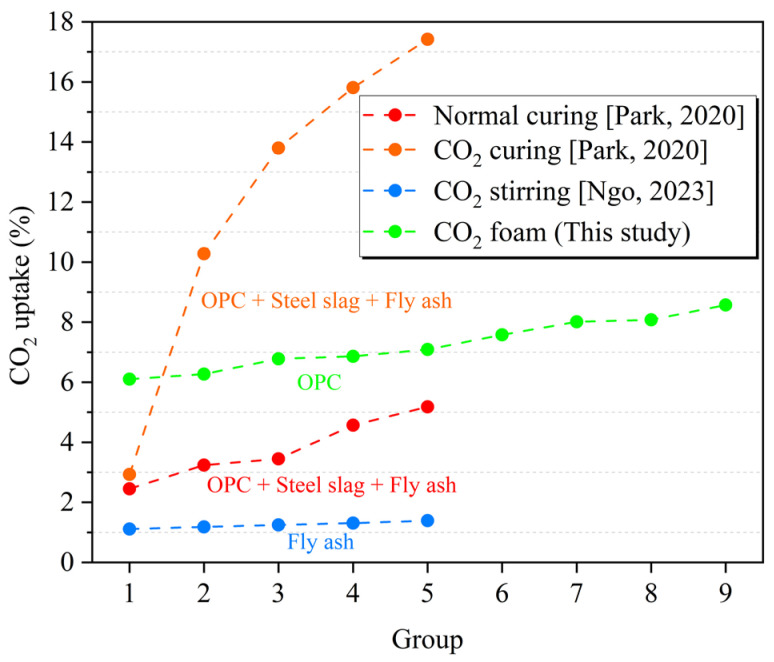
CO_2_ uptake distribution trends of various foam concrete types [5,6].

Figure 10 shows a comparison of the carbon fixation capacities of concrete materials in previous studies. The carbon fixation amount of Portland cement after natural curing was 2.45–5.18% [6], the carbon fixation amount of Portland cement after carbonization curing was 2.93%, and the carbon fixation amount of fly ash after carbonization curing was at a lower level of 1.11–1.39% [5]. After adding steel slag and other materials, it can reach 10.28–17.42% [6]. In this study, the carbon sequestration of the PC-CFC material was 6.1–8.57%. It can be seen that compared with CO_2_ curing CFC, CO_2_ foaming can improve the carbon sequestration of CFC. However, PC-CFC carbon sequestration remains insufficient compared with CFC with steel slag and other materials. There are two reasons for the low carbon fixation of PC-CFC: the insufficient stability of CO_2_ foam leads to CO_2_ escape, whereas the PC-CFC framework does not fully absorb CO_2_ during carbonation pretreatment. Generally, the maximum theoretical carbon fixation amount per ton of cement is 311 kg/ton [11]. Currently, the carbon sequestration amount of concrete buildings during their entire life cycle is 176.34 kg/ton, which is 56.7% of the theoretical value [44]. The corresponding value for PC-CFC is 62.36 kg/ton to 88.30 kg/ton, which is 20.05–28.39% of the theoretical value. This shows that the improvement direction of PC-CFC is to enhance the stability of CO_2_ foam, optimize the process parameters, and add solid waste materials such as steel slag, coal gangue, and fly ash, increasing carbon sequestration and further reducing the cost.

**Figure 11:**
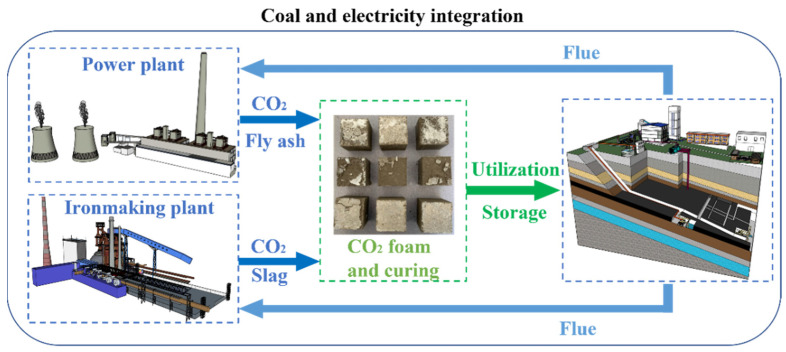
Scheme of eco-friendly production of PC-CFC from solid waste and CO_2_ to coal mines. Ironmaking plants and coal-fired power plants supply solid wastes (fly ash and slag) and CO_2_, and coal mines supply flue. Power plants and ironmaking plants source materials from the public model library.

Based on these characteristics, PC-CFC applications can operate sustainably. Figure 11 shows a schematic path of inline CO_2_ in situ storage and utilization, solid waste in situ resource utilization, and PC-CFC material production and application. Coal-fired power plants or ironmaking plants in the coal–electricity integration base can provide solid wastes (such as fly ash and slag) and CO_2_ as raw materials for PC-CFC production. The PC-CFC production process consumes a large amount of solid waste and solidifies CO_2_ in a short time. PC-CFC can be used as a backfill or thermal insulation system material to backfill the mine, which continues to provide fuel for power generation and the ironmaking industry. PC-CFC will be used for in situ treatment of CO_2_ and solid waste in coal-fired power generation, ironmaking, and mining, which provides a new way to realize low-carbon and sustainable operation of the coal–electricity integration base.

## 4. Conclusions

In this study, through carbonation pretreatment and physical foaming, mineral functional materials with adjustable porosity (65.4–77.9%), good thermal insulation performance (0.0512 W/(m·K)–0.0905 W/(m·K)), and carbon fixation potential were successfully prepared. As a new material with both thermal insulation and carbon fixation, PC-CFC provides a new feasible way for the coal integration base to achieve “carbon peak and carbon neutralization.” Based on the above research, the following conclusions were drawn:

(1) Carbonization and curing of the PC-CFC skeleton by CO_2_ will enhance the compressive strength of PC-CFC, and CO_2_ curing in pores may weaken the strength of bubbles and PC-CFC; however, PC-CFC still meets the strength requirements of the filling body in the gob.
(2) The PC-CFC exhibited good thermal insulation characteristics. Despite the problems of CO_2_ consumption and escape in the pores, PC-CFC still meets the requirements of a mine thermal insulation system. The relationship between thermal insulation and compressive strength shows that PC-CFC is expected to achieve improved thermal insulation performance and compressive strength.
(3) PC-CFC is a potentially negative carbon material, and its carbon fixation amount is 62.36–88.30 kg/ton. By adding solid waste materials to improve the process parameters and improve the preparation technology to strengthen the carbon fixation performance (up to 56.7%), the PC-CFC preparation method is expected to develop into a new CO_2_ in situ storage and utilization technology featuring CO_2_ mineralization fixation and gob filling and storage.

## Figures and Tables

**Table 1:** Chemical composition of the Portland cement (wt %).

SiO_2_	Al_2_O_3_	Fe_2_O_3_	CaO	MgO	SO_3_	Loss
24.99	8.26	4.03	51.42	3.71	2.51	3.31

**Table 2:** Mixing compositions of Portland-cement-based CO_2_ foam concrete (PC-CFC).

Specimens	Carbonation Pretreatment Time (min)	Foam Content (L)	Foaming Solution			Water/ Cement (%)
			AC-1202 (g)	HPMC (g)	Water (g)	
PC-CFC-60-4	60	4	12	24	6000	0.5
PC-CFC-60-6		6				
PC-CFC-60-8		8				
PC-CFC-90-4	90	4				
PC-CFC-90-6		6				
PC-CFC-90-8		8				
PC-CFC-120-4	120	4				
PC-CFC-120-6		6				
PC-CFC-120-8		8				

**Table 3:** Test details.

Item	Testing Age	Sample		
		Shape	Dimension	Number
Dry density	28 d	Cube	100 mm × 100 mm × 100 mm	3
Compressive strength		Cube	100 mm × 100 mm × 100 mm	3
Porosity		Cylinder	5 mm × 30 mm	1
Thermal conductivity		Slab	300 mm × 300 mm × 30 mm	3
FTIR		Powder	-	-
TG-DTG		powder	-	-

**Table 4:** Test results of PC-CFC.

Specimens	Dry Density (kg/m^3^)	28 d Compressive Strength (MPa)	CO_2_ Uptake (%)	Thermal Conductivity (W/(m·K))	Porosity (%)
PC-CFC-60-4	918	3.92	6.10	0.0905	62.61
PC-CFC-60-6	751	2.81	6.86	0.0809	70.46
PC-CFC-60-8	648	1.59	8.01	0.0732	76.82
PC-CFC-90-4	766	3.24	6.27	0.0761	70.43
PC-CFC-90-6	669	2.40	7.09	0.0739	75.26
PC-CFC-90-8	621	1.95	8.08	0.0661	77.89
PC-CFC-120-4	760	2.95	6.78	0.0720	64.40
PC-CFC-120-6	639	2.32	7.58	0.0605	71.91
PC-CFC-120-8	597	1.75	8.57	0.0512	77.90

## Data Availability

The experimental test data used to support the findings of this study are included within the article.

## References

[1] Chen W., Lu X., Lei Y., Chen J.-F. (2021). A Comparison of Incentive Policies for the Optimal Layout of CCUS Clusters in China’s Coal-Fired Power Plants Toward Carbon Neutrality. Engineering.

[2] Sang S., Yuan L., Liu S., Han S., Zheng S., Liu T., Zhou X., Wang R. (2022). Prospect of carbon neutral geological technology and its application in low carbon coal. J. China Coal Soc..

[3] Lippiatt N., Ling T.-C., Pan S.-Y. (2020). Towards carbon-neutral construction materials: Carbonation of cement-based materials and the future perspective. J. Build. Eng..

[4] Chandni T.J., Anand K.B. (2018). Utilization of recycled waste as filler in foam concrete. J. Build. Eng..

[5] Ngo I., Ma L., Zhai J., Wang Y. (2023). Enhancing fly ash utilization in backfill materials treated with CO_2_ carbonation under ambient conditions. Int. J. Min. Sci. Technol..

[6] Park B., Choi Y.C. (2021). Investigation of carbon-capture property of foam concrete using stainless steel AOD slag. J. Clean. Prod..

[7] Shah S.N., Mo K.H., Yap S.P., Yang J., Ling T.-C. (2021). Lightweight foamed concrete as a promising avenue for incorporating waste materials: A review. Resour. Conserv. Recycl..

[8] Song Q., Bao J., Xue S., Zhang P., Mu S. (2021). Collaborative disposal of multisource solid waste: Influence of an admixture on the properties, pore structure and durability of foam concrete. J. Mater. Res. Technol..

[9] Abdellatief M., Alanazi H., Radwan M.K.H., Tahwia A.M. (2022). Multiscale Characterization at Early Ages of Ultra-High Performance Geopolymer Concrete. Polymers.

[10] Tahwia A.M., Abd Ellatief M., Heneigel A.M., Abd Elrahman M. (2022). Characteristics of eco-friendly ultra-high-performance geopolymer concrete incorporating waste materials. Ceram. Int..

[11] Zhang Y., Ta X., Qin S., Hao Y. (2022). Analysis of Carbon Storage Potential of CO_2_ Foamed Concrete. Environ. Sci..

[12] Li L., Liu Q., Huang T., Li Y., Peng B. (2022). Review on CO_2_ Mineralization, Sequestration and Utilization of Cement-based Materials. Mater. Rep..

[13] Ta X., Wan Z., Zhang Y., Qin S., Zhou J. (2023). Effect of carbonation and foam content on CO_2_ foamed concrete behavior. J. Mater. Res. Technol..

[14] Ravikumar D., Zhang D., Keoleian G., Miller S., Sick V., Li V. (2021). Carbon dioxide utilization in concrete curing or mixing might not produce a net climate benefit. Nat. Commun..

[15] Lu B., Shi C., Cao Z., Guo M., Zheng J. (2019). Effect of carbonated coarse recycled concrete aggregate on the properties and microstructure of recycled concrete. J. Clean. Prod..

[16] Lu B., Shi C., Hou G. (2018). Strength and microstructure of CO2 cured low-calcium clinker. Constr. Build. Mater..

[17] Li T., Huang F., Zhu J., Tang J., Liu J. (2020). Effect of foaming gas and cement type on the thermal conductivity of foamed concrete. Constr. Build. Mater..

[18] Li T., Huang F., Li L., Zhu J., Jiang X., Huang Y. (2020). Preparation and properties of sulphoaluminate cement-based foamed concrete with high performance. Constr. Build. Mater..

[19] Ma C., Chen B. (2017). Experimental study on the preparation and properties of a novel foamed concrete based on magnesium phosphate cement. Constr. Build. Mater..

[20] Wan Z., Bi S., Zhang Y., Wang J., Wu D., Wang J. (2018). Framework of the theory and technology for simultaneous extraction of coal and geothermal resources. J. China Coal Soc..

[21] Zhang D., Ding S., Ma Y., Yang Q. (2022). Preparation and Properties of Foam Concrete Incorporating Fly Ash. Materials.

[22] Johnson Alengaram U., Al Muhit B.A., bin Jumaat M.Z., Jing M.L.Y. (2013). A comparison of the thermal conductivity of oil palm shell foamed concrete with conventional materials. Mater. Des..

[23] Wu D., Wan Z., Zhang H., Zhang Y., Wang Z., Lu N. (2019). Experimental Study on New Thermal Insulating Material for Mine. Bull. Chin. Ceram. Soc..

[24] Nambiar E.K., Ramamurthy K. (2006). Models relating mixture composition to the density and strength of foam concrete using response surface methodology. Cem. Concr. Compos..

[25] Liu X. (2019). Research on the Mechanism of CO_2_ Absorption by Fresh Cement-Based Materials on Their Hydration and Hardening. Master’s Thesis.

[26] Lv M., Wang S., Zhai Z., Luo X., Jing Z. (2016). Comparative investigation of the static and dynamic properties of CO_2_ foam and N_2_ foam. Can. J. Chem. Eng..

[27] Parra J.G., Domínguez H., Aray Y., Iza P., Zarate X., Schott E. (2019). Structural and interfacial properties of the CO_2_-in-water foams prepared with sodium dodecyl sulfate (SDS): A molecular dynamics simulation study. Colloids Surf. A Physicochem. Eng. Asp..

[28] Yan D., Lu J., Sun Y., Wang T., Meng T., Zeng Q., Liu Y. (2021). CO2 Pretreatment to Aerated Concrete with High-Volume Industry Wastes Enables a Sustainable Precast Concrete Industry. ACS Sustain. Chem. Eng..

[29] Moon E.-J., Choi Y.C. (2019). Carbon dioxide fixation via accelerated carbonation of cement-based materials: Potential for construction materials applications. Constr. Build. Mater..

[30] Liu K., Zhang J., Tian X., Huang D., Peng H. (2023). Improving carbon sequestration, mechanical properties and thermal insulation of RMFC by foaming with H_2_O_2_ and carbonization curing. Mater. Rep..

[31] Xue Q., Zhang L., Mei K., Wang L., Wang Y., Li X., Cheng X., Liu H. (2022). Evolution of structural and mechanical properties of concrete exposed to high concentration CO_2_. Constr. Build. Mater..

[32] Amran Y.M., Farzadnia N., Ali A.A. (2015). Properties and applications of foamed concrete; a review. Constr. Build. Mater..

[33] Kupwade-Patil K., Palkovic S.D., Bumajdad A., Soriano C., Büyüköztürk O. (2018). Use of silica fume and natural volcanic ash as a replacement to Portland cement: Micro and pore structural investigation using NMR, XRD, FTIR and X-ray microtomography. Constr. Build. Mater..

[34] Pan X., Shi Z., Shi C., Hu X., Wu L. (2016). Interactions between inorganic surface treatment agents and matrix of Portland cement-based materials. Constr. Build. Mater..

[35] Ashraf W., Olek J. (2016). Carbonation behavior of hydraulic and non-hydraulic calcium silicates: Potential of utilizing low-lime calcium silicates in cement-based materials. J. Mater. Sci..

[36] Ashraf W., Olek J., Atakan V. A comparative study of the reactivity of calcium silicates during hydration and carbonation reactions. Proceedings of the 14th International Congress on Cement Chemistry.

[37] Lu B. (2020). The Harding Behaviour of CO_2_-Cured Portland Cement and Post Hydration. Ph.D. Thesis.

[38] Villain G., Thiery M., Platret G. (2007). Measurement methods of carbonation profiles in concrete: Thermogravimetry, chemical analysis and gammadensimetry. Cem. Concr. Res..

[39] Thiery M., Villain G., Dangla P., Platret G. (2007). Investigation of the carbonation front shape on cementitious materials: Effects of the chemical kinetics. Cem. Concr. Res..

[40] Belem T., Benzaazoua M. (2008). Design and Application of Underground Mine Paste Backfill Technology. Geotech. Geol. Eng..

[41] Qi C., Fourie A. (2019). Cemented paste backfill for mineral tailings management: Review and future perspectives. Miner. Eng..

[42] Zhang X., Yang Q., Shi Y., Zheng G., Li Q., Chen H., Cheng X. (2020). Effects of different control methods on the mechanical and thermal properties of ultra-light foamed concrete. Constr. Build. Mater..

[43] Luo K., Li J., Lu Z., Wang L., Deng X., Hou L., Jiang J. (2021). Preparation and performances of foamed hydraulic lime. Constr. Build. Mater..

[44] Chen B., Harp D.R., Lin Y., Keating E.H., Pawar R.J. (2018). Geologic CO_2_ sequestration monitoring design: A machine learning and uncertainty quantification based approach. Appl. Energy.

